# Development and internal validation of a risk stratification scoring system for identifying gram-negative ESBL-positive acute cholangitis: A retrospective cohort study

**DOI:** 10.1371/journal.pone.0345150

**Published:** 2026-03-17

**Authors:** Phoomjai Sornsenee, Chanchanok Chaochuensuk, Duangkiat Sapsong, Kanyakorn Khongchuy, Panitsara Raksorn, Paisit Kangton, Phirada Khongrueang, Suppachai Choengdee, Suphajit Nuypud, Teerapob Wetchasit, Thanet Oonthae, Tanawat Pattarapuntakul, Nisa Netinatsunton, Jaksin Sottisuporn, Thanawin Wong

**Affiliations:** 1 Department of Family Medicine and Preventive Medicine, Faculty of Medicine, Prince of Songkla University, Hat Yai, Songkhla, Thailand; 2 Faculty of Medicine, Prince of Songkla University, Hat Yai, Songkhla, Thailand; 3 Gastroenterology and Hepatology Unit, Division of Internal Medicine, Faculty of Medicine, Prince of Songkla University, Hat Yai, Songkhla, Thailand; 4 NKC Institute of Gastroenterology and Hepatology, Faculty of Medicine, Prince of Songkla University, Hat Yai, Songkhla, Thailand; Fayetteville State University, UNITED STATES OF AMERICA

## Abstract

**Background:**

Acute cholangitis, a severe biliary infection that is frequently caused by gram-negative pathogens, presents health challenges owing to the increasing prevalence of antimicrobial resistance, particularly among extended-spectrum beta–lactamase (ESBL)-producing bacteria. Accurate risk stratification of patients with extended-spectrum beta-lactamase–producing bacteria is crucial for optimizing antimicrobial therapies.

**Aims:**

This study aimed to develop and internally validate a risk stratification scoring system for identifying ESBL-producing bacteria in patients with acute cholangitis, with the goal of supporting clinical risk stratification.

**Methods:**

This retrospective cohort analysis included adult patients with acute cholangitis admitted between 2019 and 2023. Patients were excluded if they had incomplete medical records, missing microbiological data, or non-adherence to the Tokyo Guidelines 2018 diagnostic criteria. Predictors of positivity for ESBL-producing bacteria were identified using multivariate logistic regression and integrated into a scoring system, and the performance metrics were evaluated.

**Results:**

A total of 303 patients with positive blood or bile cultures were included in the analysis, of whom 111 (36.6%) had ESBL-positive cholangitis. Independent predictors of positivity for ESBL-producing bacteria included prior antibiotic treatment (3.5 points), chills with rigors (2.5 points), alanine aminotransferase levels <400 U/L (3.5 points), hematocrit levels <27% (2.5 points), and alkaline phosphatase levels >300 U/L (2.0 point). At cutoff scores of 7.5 and 11, the scoring system demonstrated sensitivity of 56% and 16%, specificity of 75% and 98%, positive predictive values of 56% and 82%, and overall accuracy of 68% and 70%, respectively.

**Conclusions:**

This preliminary scoring system demonstrated high specificity at higher cutoffs, supporting its use as a rule-in tool for identifying patients at high risk of ESBL-producing bacteria in acute cholangitis. However, its moderate sensitivity limits its role as a rule-out strategy. This preliminary internally validated model should not be used to guide routine clinical decision-making without external multicenter validation, and any application must consider local resistance patterns and patient context.

## Introduction

Acute cholangitis, which is also known as ascending cholangitis, is a serious infection of the biliary tract that stems from simultaneous bile duct obstruction and bacterial colonization. Obstructions, which frequently result from gallstones, strictures, or tumors, lead to bile stasis and create conditions conducive to bacterial growth [1]. This pathological condition is associated with high mortality rates, especially in severe cases, where the mortality rate may increase to 30% [2]. Acute cholangitis is caused by gram-negative bacilli in approximately 60–90% of cases, including *Escherichia coli*, *Klebsiella pneumoniae*, and Enterobacter species, which can ascend from the duodenum to the biliary tree [3–5]. The foundation of treatment involves the concurrent administration of antimicrobial therapy with biliary drainage. The primary goal of antimicrobial therapy for acute cholangitis is to mitigate systemic sepsis and prevent intrahepatic abscess formation and consequently permitting elective biliary drainage, instead of emergency procedures [6,7].

The prevalence of extended-spectrum beta-lactamase (ESBL)-producing pathogens has increased over time, particularly among patients with prior biliary procedures, and has affected a wide range of bacterial species, including major pathogens implicated in biliary infections, such as *Escherichia coli* and *Klebsiella pneumoniae* [5,7–10]. Detection rates of ESBL-producing *Escherichia coli* isolates in clinical settings vary considerably, ranging from 10–30% in community-acquired cases to as high as 50% in hospital-acquired cases [7–9]. These organisms can hydrolyze broad-spectrum beta-lactam antibiotics and thereby lead to higher treatment failure rates, prolonged hospitalization, increased healthcare costs, and elevated morbidity and mortality rates [11, 12]. Notably, there is high antimicrobial resistance in nosocomial infections and patients with a history of biliary procedures, which can introduce and promote colonization by resistant strains [11,13]. This variability highlights the need for developing targeted therapeutic strategies.

The Tokyo Guidelines 2018 (TG2018) are standard guidelines that provide a framework for diagnosing and grading the severity of the condition and help clinicians determine the appropriate level of intervention, which ranges from medical management to endoscopic or surgical procedures for biliary decompression [6]. Despite these guidelines, current strategies do not provide methods for the early identification of patients at risk of infections caused by ESBL-producing bacteria. Guidance for updating the local antibiogram and choosing the antimicrobial agent depends on the grading of disease severity.

Therefore, a clinically practical and internally validated risk-stratification score is needed to estimate the probability of ESBL–producing bacteria among patients with acute cholangitis. Such a tool may help identify patients at increased risk and may support earlier selection of appropriate empiric antimicrobial therapy. To our knowledge, no validated prediction model currently exists to specifically estimate ESBL positivity in this clinical setting. Accordingly, we developed and internally validated a predictive scoring system to assist risk stratification for ESBL-producing pathogens in gram-negative acute cholangitis.

## Materials and methods

### Study design

This was a retrospective cohort study conducted at Songklanagarind Hospital, which is the only university hospital in Southern Thailand, and included adult patients (aged 18 years and above) diagnosed with acute cholangitis from January 2019 to December 2023. Patients were excluded if they did not meet the TG2018 diagnostic criteria, had incomplete medical records, or lacked blood or bile culture sampling during their admission. Data were accessed on 20/09/2024 for research purposes. All patients with acute cholangitis were managed according to the sepsis protocol. Hemocultures were obtained prior to the initiation of antimicrobial therapy. Empirical antimicrobial agents were administered immediately afterward, selected based on local susceptibility patterns and in accordance with the TG2018 guidelines. Biliary drainage was performed by expert endoscopists, with the timing of the procedure determined by disease severity as outlined in the guideline [14]. Following successful biliary cannulation and prior to cholangiography, bile samples were aspirated for microbial culture. Bile cultures were thus collected after the initiation of antimicrobial therapy. Phenotypic assays were utilized for the detection of antimicrobial resistance. All bacterial isolates underwent phenotypic antimicrobial susceptibility testing using standard protocols to determine ESBL production. The study protocol was approved by the Human Research Ethics Committee (HREC), Faculty of Medicine, Prince of Songkla University (Approval Number: REC. REC.67-395-9-1, approval date: 18 September 2024). This study was conducted in compliance with the principles of the Declaration of Helsinki. As this was a retrospective study that utilized computerized data, the requirement of informed consent was waived. All electronic data obtained from the Division of Digital Innovation and Data Analytics database (DIDA) were securely stored on servers that were accessible only through the institutional email account of the principal investigator. Requests for access or queries regarding data management can be directed to the principal investigator via email. Physical data storage devices were kept in a secure, locked file cabinet that was accessible only to authorized personnel. In accordance with the HREC requirements, data will be retained for 3 years after study completion and, thereafter, all electronic data will be permanently erased, and physical records will be destroyed in accordance with the institutional policies on data disposal.

### Data collection

Data were extracted from the hospital’s electronic health record system and subsequently reviewed against detailed clinical notes to ensure accuracy. Selected variables, particularly those requiring clinical interpretation, were manually verified against original medical records by investigators to confirm data correctness. Patient identification was performed using specific ICD-10 codes, such as K80.3 (calculus of the bile duct with cholangitis), K83.1 (obstruction of bile duct passage), and C22.1 (intrahepatic bile duct carcinoma). After obtaining the necessary permission from the DIDA, relevant data, including patient demographics (age, sex), medical history (comorbidities, prior hospitalization, prior antimicrobial use, and chemotherapy), clinical symptoms (fever, rigors/chills), severity of cholangitis according to the Tokyo Guidelines, baseline blood chemistry (hematocrit, albumin, alanine aminotransferase (ALT), alkaline phosphatase (ALP), bilirubin, etc.), and microbiological findings (blood and bile culture results), were extracted from the hospital database. Each record was rigorously reviewed to ensure its validity for inclusion, highlighting the identification of ESBL-producing bacteria. Verified data points were anonymized and securely transferred for statistical analysis.

### Statistical analysis

**Fig 1:**
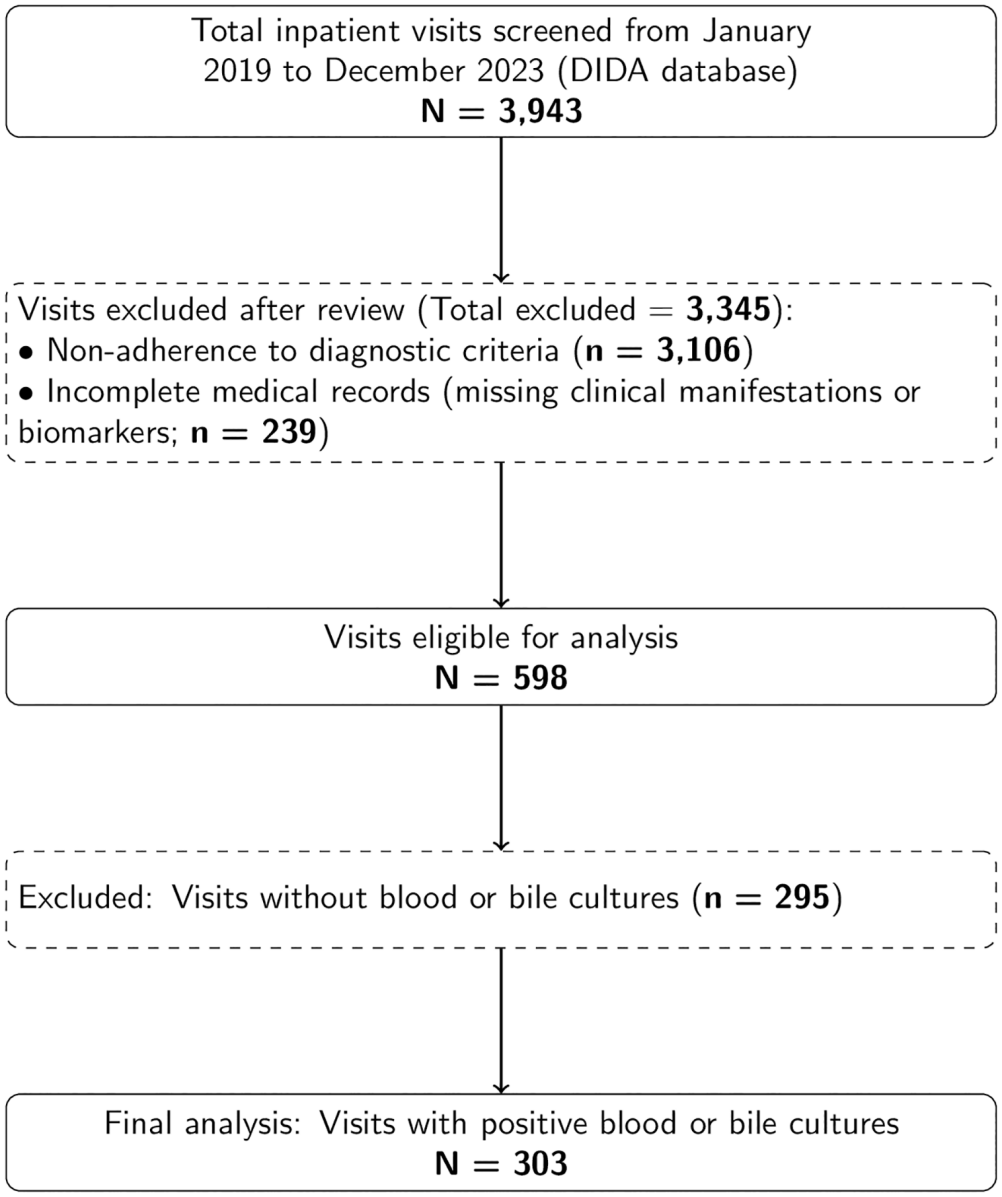
Study flowchart depicting patient inclusion criteria.

Data were anonymized, organized in Microsoft Excel, and subsequently transferred to R (version 4.2.2; R Foundation for Statistical Computing, Vienna, Austria) for statistical analysis. Records with missing data in key predictors or outcome variables were excluded during cohort assembly, as illustrated in the study flow diagram (Fig 1). The final analyses were performed using complete cases only, without data imputation; patients with missing data in any candidate predictors were excluded from model construction.

The independent variables included patient demographics (age, sex), prior biliary procedures, and antimicrobial use within 30 days before admission, as well as clinical severity of cholangitis as defined by the Tokyo Guidelines 2018, which were considered in the multivariable model to account for potential confounding. Microbiological analyses focused on detecting ESBL-producing organisms in bile or blood cultures, highlighting the presence of resistant strains. Additionally, data on antibiotic use were meticulously documented and analyzed to assess patterns of resistance.

Descriptive statistics were used to summarize the patient characteristics. Continuous variables were reported as median with interquartile range (IQR), which represents the inclusive 25th and 75th percentiles, or as mean with standard deviation (SD), depending on data distribution. Continuous variables were converted into categorical variables using cut points derived from the observed distribution of each variable. The Mann–Whitney U test was applied for continuous variables, while the Chi-square test or Fisher’s exact test was used for categorical variables. Univariate logistic regression was first performed to screen candidate predictors of ESBL positivity. Variables with p < 0.20 in univariate analysis were entered into the multivariable logistic regression model. For continuous predictors, clinically plausible ranges and commonly used thresholds in routine practice were initially explored. Final cut points were subsequently determined using receiver operating characteristic (ROC) analysis, considering sensitivity, specificity, likelihood ratios, and the Youden index to optimize discrimination within the study dataset. Continuous variables were categorized to facilitate construction of a practical bedside scoring system. The direction and magnitude of associations in the multivariable model were then reviewed to confirm consistency with the selected cut points and their contribution to overall model performance. A higher cut-off was additionally defined based on predicted probability to identify patients with a high certainty of ESBL positivity. Model adequacy was evaluated by assessing multicollinearity using variance inflation factors, calibration using the Hosmer–Lemeshow goodness-of-fit test, and overall fit using Nagelkerke’s pseudo-R².

The discriminative and calibration performance of the final model was evaluated using the area under the receiver operating characteristic curve (AUC-ROC). The scoring system was derived from the regression coefficients of the final logistic regression model (log-odds scale), which were divided by the smallest absolute coefficient retained in the final model (common reference) to preserve their relative contributions. The resulting values were then rounded to the nearest 0.5 to enhance clinical usability and ease of bedside calculation, resulting in an additive point-based scoring system. The composite score was subsequently evaluated for overall discrimination using AUC and for calibration using the Hosmer–Lemeshow goodness-of-fit test. Internal validation was conducted using bootstrapping with 500 resamples to evaluate model stability, while calibration was further examined by comparing predicted and observed outcomes.

## Results

Fig 1 depicts the study flow. A total of 3,943 inpatient visits were screened between January 2019 and December 2023. After excluding 3,106 visits that did not meet the diagnostic criteria, an additional 239 visits were excluded because essential clinical manifestations or biomarker data were incomplete. Among the remaining visits, 295 were further excluded due to the absence of blood or bile culture results. To preserve independence of observations, only one eligible episode per patient was included in the final analysis. The resulting analytic cohort therefore consisted of 303 unique patients.

The demographic and clinical characteristics of the patients are summarized in Table 1. 111 (36.6%) patients were ESBL-positive. The median age and sex distribution were comparable between groups. Chills and rigors were significantly more prevalent in the ESBL-positive group. Prior chemotherapy, antimicrobial treatment, and hospitalization within 30 days were also more frequent in ESBL-positive patients. Regarding laboratory findings, this group had significantly lower hematocrit and serum albumin levels, and lower ALT, whereas ALP levels were significantly higher. A greater proportion of ESBL-positive patients also had hematocrit <27% and albumin <2.5 g/dL.

**Table 1:** ESBL-status-stratified clinicodemographic and laboratory characteristics of patients with acute cholangitis.

Parameters	Total (n = 303)	ESBL-negative (n = 192)	ESBL-positive (n = 111)	*p*-value
Age, years, median (IQR)	70 (58–78)	70 (58.8–79)	70 (56–76.5)	0.256
Male sex, n (%)	164 (51.1)	100 (52.1)	64 (57.7)	0.348
**Clinical Manifestation**, n (%)				
Fever	184 (60.7)	116 (60.4)	68 (61.3)	0.885
Chills/ Rigors	120 (39.6)	64 (33.3)	56 (50.5)	0.004*
**Previous clinical status**, n (%)				
Comorbidities				
Diabetes mellites	69 (22.8)	48 (25)	21 (18.9)	0.224
Hypertension	130 (42.9)	85 (44.3)	45 (40.5)	0.527
Chronic liver disease	40 (13.2)	22 (11.5)	18 (16.2)	0.238
Cardiovascular disease	45 (45.9)	29 (15.1)	16 (14.4)	0.871
Chronic kidney disease	35 (11.6)	21 (10.9)	14 (12.6)	0.660
Prior antibiotic treatment, n (%)	52 (17.2)	20 (10.4)	32 (28.8)	<0.001*
Prior chemotherapy within 30 days, n (%)	24 (7.9)	10 (5.2)	14 (12.6)	0.026*
Prior hospitalization within 30 days, n (%)	61 (20.1)	30 (15.6)	31 (27.9)	<0.001*
**Cholangitis-related Factors**, n (%)				
Proportion of definite diagnosis	234 (77.2)	150 (78.1)	84 (75.7)	0.624
Post-ERCP cholangitis	20 (6.6)	11 (5.7	9 (8.1)	0.424
Severity				0.200
Grade I mild	95 (31.4)	64 (33.3)	31 (27.9)	
Grade II moderate	102 (33.7)	68 (35.4)	34 (30.6)	
Grade III severe	106 (35.0)	60 (31.2)	46 (41.4)	
Organ dysfunction				
Hematological	23 (7.6)	15 (7.8)	8 (7.2)	0.848
Hepatic	63 (20.8)	34 (17.7)	29 (26.1)	0.084
Renal	33 (10.9)	17 (8.9)	16 (14.4)	0.138
Respiratory	6 (2)	2 (1)	4 (3.6)	0.147
Neurological	9 (3)	6 (3.1)	3 (2.7)	0.835
Cardiovascular	16 (5.3)	8 (4.2)	8 (7.2)	0.260
Etiologies				0.071
Choledocholithiasis	160 (52.8)	108 (56.2)	52 (46.8)	
Benign distal	28 (9.2)	19 (9.9)	9 (8.1)	
Malignant hilar	33 (10.9)	18 (9.4)	15 (13.5)	
Malignant distal	14 (4.6)	7 (3.6)	7 (6.3)	
Unidentification	68 (22.4)	40 (20.8)	28 (25.2)	
**Laboratory**				
**Continuous data**				
White blood cell count, median (IQR)	12500 (9385–16740)	12590 (9930–16392.5)	12060 (8255–17665)	0.661
PMN, median (IQR)	86 (78.3–90.3)	86 (76.9–90.3)	85 (79.8–90.7)	0.799
Hematocrit, mean (SD)	33.3 (7.1)	34.5 (7)	31.2 (6.7)	<0.001*
Platelet, median (IQR)	217000 (159500–297500)	214500 (159500–278500)	226000 (160500–308500)	0.363
INR, median (IQR)	1.2 (1.1–1.4)	1.3 (1.1–1.5)	1.2 (1.1–1.4)	0.057
PT, median (IQR)	27.5 (24.6–31.5)	27 (24–31)	28.4 (25.6,32.1)	0.020*
GFR, median (IQR)	80 (53.5–98)	76 (53.8–94.2)	83 (53.5–104)	0.213
AST, median (IQR)	137 (74.5–262.5)	158.5 (88–287)	118 (62–210.5)	<0.001*
ALT, median (IQR)	110 (53.5–216)	139.5 (77–254.5)	82 (45–152)	<0.001*
ALP, median (IQR)	270 (174–446)	253.5(166.8–377.8)	316 (192.5–599)	0.003*
Total bilirubin, median (IQR)	3.8 (2.3–7.4)	3.8 (2.4–6.8)	4 (2.2–11.8)	0.547
Direct bilirubin, median (IQR)	3 (1.8–5.8)	3 (1.8–5.1)	2.9 (1.7–9.2)	0.456
Albumin, median (IQR)	3.4 (2.8–3.9)	3.6 (3.1–4)	3.2 (2.6–3.7)	<0.001*
**Categorical data**, n (%)				
Albumin <2.5 g/dL	30 (9.9)	12 (6.2)	18 (16.2)	0.007*
ALP > 300 IU/L	128 (42.2)	69 (35.9)	59 (53.2)	0.004*
ALT < 400 IU/L	278 (91.7)	170 (88.5)	108 (97.3)	0.014*
Hematocrit <27%	67(22.1)	30 (15.6)	37 (33.3)	<0.001*

ESBL: extended-spectrum beta-lactamase, IQR: interquartile range, PMN: polymorphonuclear leukocyte, HCT: hematocrit, INR: international normalized ratio, PT:, GFR: glomerular filtration rate, AST: aspartate transferase, ALT: alanine aminotransferase, ALP: alkaline phosphatase, ERCP: Endoscopic-retrograde cholangiopancreatography.

*p < 0.05 indicated statistically significant differences.

The clinical outcomes, microbiological results, empirical antibiotics used, and resistance profiles are summarized in Tables 2–4. ESBL-positive patients had a significantly longer median hospital stay. The rate of early clinical resolution within 72 hours was lower in the ESBL-positive group, although this difference did not reach statistical significance. Recovery and mortality outcomes at discharge were comparable between groups. Concerning microbiological findings, blood cultures were more frequently positive in ESBL-negative patients, while bile cultures were more often positive in ESBL-positive patients.

**Table 2:** Clinical outcomes and microbiological findings in patients with acute cholangitis.

Clinical Outcomes	Total (N = 303)	Non-ESBL isolates (n = 192)	ESBL isolates (n = 111)	*P*-value
Length of stay (days), median (IQR)	7 (5,11)	6 (4,9.2)	8 (5,13.5)	<0.001*
Clinical resolution within 72 h	261 (86.1)	171 (89.1)	90 (81.1)	0.078
In-hospital mortality rate	18 (5.9)	12 (6.2)	6 (5.4)	0.962
**Microbial culture-positive results (blood or bile)**				
Blood culture positive	195 (64.4)	150 (78.1)	45 (40.5)	<0.001*
Bile culture positive	176 (58.1)	99 (51.6)	77 (69.4)	<0.001*
Both blood and bile cultures positive	68 (22.4)	47 (24.5)	21 (18.9)	0.330

ESBL: extended-spectrum beta-lactamase, IQR: interquartile range.

*p < 0.05, statistically significant differences.

**Table 3:** Empirical antimicrobials used in patients with acute cholangitis.

Antimicrobial class	n (%)
Cephalosporins (e.g., ceftriaxone)	137 (45.2)
β-lactam/β-lactamase inhibitors (e.g., piperacillin–tazobactam)	70 (23.1)
Carbapenems (e.g., meropenem)	53 (17.5)
Quinolones (e.g., ciprofloxacin)	13 (4.3)
Penicillins (e.g., ampicillin)	3 (1.0)
Aminoglycosides (e.g., gentamicin)	2 (0.7)
Other antibiotics	3 (1.0)
Combination therapy	22 (7.3)

Note: This table describes the empirical antimicrobial choices that were made by clinicians based on local susceptibility pattern and TG 2018 guidelines when patients were initially diagnosed with acute cholangitis. Other monotherapy included glycopeptide, clindamycin, and cotrimoxazole. Combination therapy included regimens such as cephalosporin plus metronidazole and other multi-drug combinations.

**Table 4:** Distribution of Gram-negative pathogens isolated from blood and bile samples in patients with acute cholangitis.

Pathogens	Blood (n = 195), n (%)		Bile (n = 176), n (%)	
	Non-ESBL producing	ESBL producing	Non-ESBL producing	ESBL producing
*Escherichia coli*	86 (44.1)	34 (17.4)	59 (33.5)	36 (20.5)
*Klebsiella pneumoniae*	37 (19.0)	11 (5.6)	37 (21.0)	41 (23.3)
*Aeromonas* spp.	7 (3.6)	0 (0.0)	19 (10.8)	0 (0.0)
*Enterobacter* spp.	5 (2.6)	0 (0.0)	18 (10.2)	0 (0.0)
*Pseudomonas* spp.	2 (1.0)	0 (0.0)	16 (9.1)	0 (0.0)
*Acinetobacter baumannii*	5 (2.6)	0 (0.0)	7 (4.0)	0 (0.0)
*Stenotrophomonas maltophilia*	1 (0.5)	0 (0.0)	6 (3.4)	0 (0.0)
*Klebsiella aerogenes*	2 (1.0)	0 (0.0)	4 (2.3)	0 (0.0)
*Citrobacter* spp.	2 (1.0)	0 (0.0)	4 (2.3)	0 (0.0)
*Proteus* spp.	0 (0.0)	0 (0.0)	5 (2.8)	0 (0.0)
*Morganella morganii*	1 (0.5)	0 (0.0)	2 (1.1)	0 (0.0)
*Klebsiella oxytoca*	0 (0.0)	0 (0.0)	3 (1.7)	0 (0.0)
*Salmonella non-typhi*	1 (0.5)	0 (0.0)	0 (0.0)	0 (0.0)
*Vibrio fluvialis*	1 (0.5)	0 (0.0)	0 (0.0)	0 (0.0)
*Rhizobium radiobacter*	0 (0.0)	0 (0.0)	1 (0.6)	0 (0.0)
*Shewanella algae*	0 (0.0)	0 (0.0)	1 (0.6)	0 (0.0)

ESBL: extended-spectrum beta-lactamase.

Table 3 summarizes the empirical antibiotic choices, with ceftriaxone being the most frequently prescribed agent, followed by piperacillin–tazobactam and meropenem. Tables 4 and S1 show the microbial and resistance profiles. *E. coli* was the most common pathogen in both ESBL-negative and ESBL-positive groups, followed by *K. pneumoniae*. ESBL-positive isolates demonstrated complete resistance to third-generation cephalosporins, and high resistance to ciprofloxacin and piperacillin–tazobactam. In contrast, resistance to carbapenems remained low among non-ESBL isolates but was remarkably higher in ESBL-positive *K. pneumoniae*.

**Fig 2:**
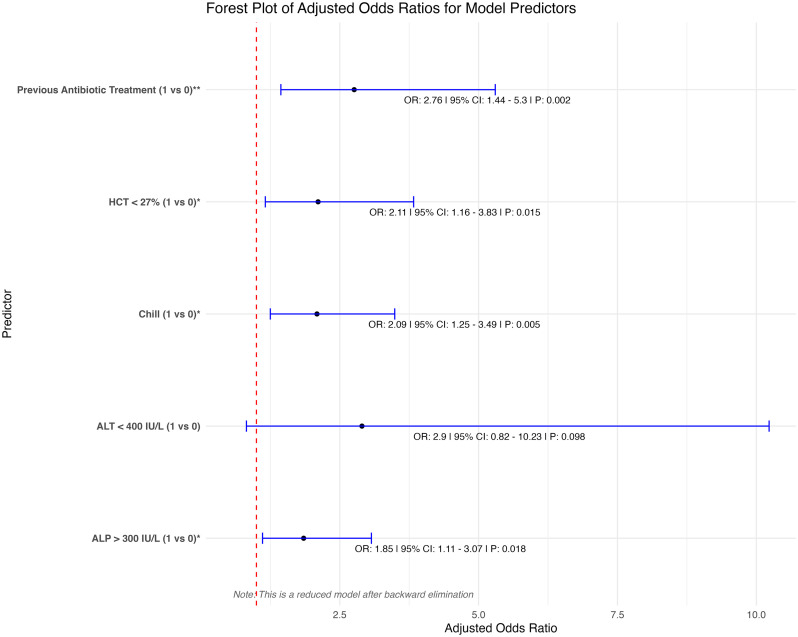
Multivariate logistic regression analysis of predictors for ESBL-positive status in acute cholangitis.

Fig 2 presents the results of the multivariate logistic regression analysis to identify independent predictors of ESBL-positive status in patients with acute cholangitis. The analysis revealed significant predictors, including prior antimicrobial treatment, chills, ALT < 400 U/L, hematocrit <27%, and ALP > 300 U/L. These predictors highlight the clinical and laboratory features most strongly associated with ESBL positivity. The regression model demonstrated no evidence of multicollinearity, adequate calibration on the Hosmer–Lemeshow test, and a Nagelkerke’s pseudo-R² of 0.179, indicating modest explanatory power. Despite this, the model showed the ability to discriminate between ESBL-positive and ESBL-negative cases.

Table 5 presents the final multivariable logistic regression model and the corresponding scoring system developed to stratify the risk of ESBL-positive status among patients with acute cholangitis. Five predictors were retained in the final model: prior antimicrobial use, chills, ALT < 400 U/L, hematocrit <27%, and ALP > 300 U/L.

**Table 5:** Final multivariable logistic regression model and assigned scores for ESBL risk stratification in acute cholangitis.

Predictor	β coefficient (log-odds)	SE	Adjusted OR	95% CI	p-value	Assigned score
**Chills (present vs absent)**	0.738	0.261	2.09	1.25–3.49	0.005	2.5
**ALT < 400 IU/L (vs ≥ 400 IU/L)**	1.064	0.643	2.90	0.82–10.23	0.098	3.5
**Hematocrit <27% (vs ≥ 27%)**	0.744	0.305	2.11	1.16–3.83	0.015	2.5
**Prior antibiotic use (yes vs no)**	1.016	0.332	2.76	1.44–5.30	0.002	3.5
**ALP > 300 IU/L (vs ≤ 300 IU/L)**	0.613	0.260	1.85	1.11–3.07	0.018	2.0
**Intercept**	−2.494	0.642	—	—	<0.001	—

ESBL: extended-spectrum beta-lactamase CI: confidence interval, LHR: likelihood ratio, ALT: alanine aminotransferase, ALP: alkaline phosphatase.

Note: The presented model represents the reduced multivariable logistic regression model derived from an initial full model including candidate predictors with univariable p < 0.20, namely chills/rigors, prior antibiotic treatment, prior chemotherapy, prior hospitalization, severity grade, hepatic dysfunction, renal dysfunction, respiratory dysfunction, etiology category, hematocrit, INR, PT, AST, albumin <2.5 g/dL, ALP > 300 IU/L, ALT < 400 IU/L, and hematocrit <27%. β coefficients represent unscaled log-odds estimates from the final multivariable logistic regression model. Assigned scores were derived from these coefficients by rescaling to preserve relative contributions and rounding to the nearest 0.5 to facilitate clinical application. For all binary predictors, the absence or opposite category was used as the reference.

**Fig 3:**
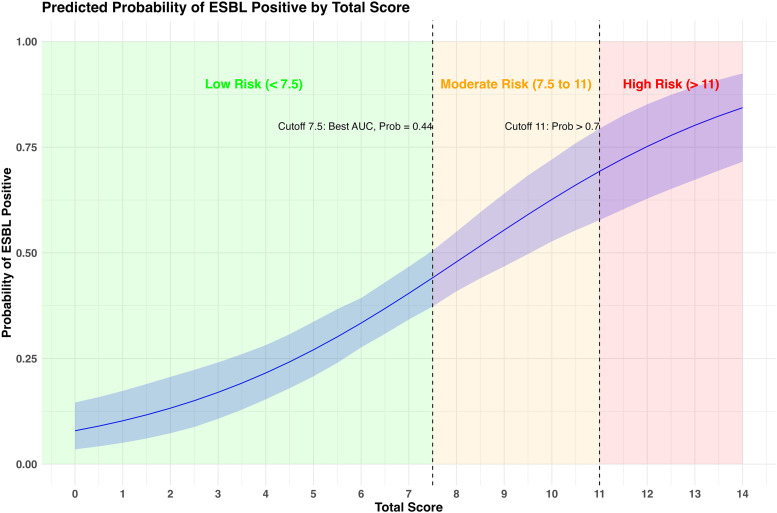
Visualization of predicted probability for ESBL-positive status using the scoring system.

The discriminative performance of the composite score, summarized in Table 6, increased with higher cutoffs. At a cutoff ≥7.5, the model demonstrated moderate discrimination, with a sensitivity of 0.56 (95% CI, 0.46–0.66) and a specificity of 0.75 (95% CI, 0.68–0.81), supporting its use for early risk stratification. In contrast, at a cutoff ≥11, specificity increased substantially to 0.98 (95% CI, 0.96–1.00), accompanied by a marked rise in the positive likelihood ratio to 7.78 (95% CI, 2.85–35.31), albeit with reduced sensitivity. This pattern reflects the trade-off between case detection (false-negative classification) and diagnostic certainty (false-positive classification), allowing clinicians to tailor score interpretation according to the intended clinical context. Fig 3 further illustrates the relationship between the composite score and the predicted probability of ESBL positivity across the full score range.

**Table 6:** Performance metrics of the scoring system for predicting ESBL-positivity status in patients with acute cholangitis.

Performance metric	Cutoff ≥7.5 (95% CI)	Cutoff ≥11 (95% CI)
Sensitivity	0.56 (0.46–0.66)	0.16 (0.09–0.24)
Specificity	0.75 (0.68–0.81)	0.98 (0.96–1.00)
PPV	0.56 (0.46–0.66)	0.82 (0.61–0.96)
NPV	0.75 (0.67–0.81)	0.67 (0.61–0.72)
LHR+	2.19 (1.55–3.12)	7.78 (2.85–35.31)
LHR−	0.59 (0.45–0.75)	0.86 (0.78–0.93)

ESBL: extended-spectrum beta-lactamase, PPV: positive predictive value, NPV: negative predictive value.

## Discussion

Our study developed and internally validated a scoring system to predict ESBL-positivity in patients with acute cholangitis. At a cutoff score of ≥7.5, the scoring system yielded a sensitivity of 0.56 (95% CI: 0.46–0.66) and a specificity of 0.75 (95% CI: 0.68–0.81), indicating moderate ability to identify true ESBL-positive cases and good ability to correctly exclude ESBL-negative cases. However, the modest sensitivity at this threshold reflects that 44% of ESBL-positive cases would be misclassified as low-risk (false negatives). In clinical practice, this limitation has important implications: patients with scores below 7.5 may still harbor ESBL-producing organisms, particularly in settings with high local ESBL prevalence or in individuals with additional unmeasured risk factors such as recent healthcare exposure or indwelling biliary devices. At the higher cutoff of ≥11, specificity improved markedly to 0.98 (95% CI: 0.94–0.99), with a positive predictive value of 0.82, supporting high-certainty identification of ESBL-positive cases. At this threshold, only 2% of ESBL-negative patients are misclassified as high-risk (false positives). While this may lead to unnecessary carbapenem use in a small subset of patients, the high specificity and positive likelihood ratio justify empirical carbapenem therapy in this group, particularly when timely culture results are unavailable or when the clinical severity warrants coverage of resistant pathogens. The trade-off between false negatives at lower cutoffs and false positives at higher cutoffs reflects a fundamental tension in antimicrobial stewardship, balancing adequate empirical coverage with the need to minimize unnecessary broad-spectrum antibiotic use. To improve clinical interpretability and bedside applicability, regression coefficients expressed on a log-odds scale were transformed into an additive point-based scoring system, with rounding to the nearest 0.5 applied to facilitate ease of use while preserving the relative contribution of each predictor. Similar scores across different predictors reflect comparable effect sizes after rescaling and rounding, rather than arbitrary assignment. This approach allows flexible risk stratification according to institutional priorities and local resistance epidemiology. In settings with moderate-to-high ESBL prevalence, the higher cutoff may be preferred to identify patients most likely to benefit from carbapenem therapy, whereas in lower-resistance settings, the lower cutoff may help reserve carbapenems for higher-risk patients while permitting narrower-spectrum therapy in most cases. The scoring system incorporates both clinical predictors including chills (2.5 points) and prior antimicrobial treatment (3.5 points), and laboratory variables including ALT < 400 U/L (3.5 points), hematocrit <27% (2.5 points), and ALP > 300 U/L (2.0 points), providing a practical framework for guiding empirical antimicrobial selection in the context of rising resistance.

The current study confirmed that E. coli was the most common pathogen (61.5% of blood and 54% of bile isolates), followed by *K. pneumoniae* (24.6% of blood and 44.3% of bile isolates), which is consistent with previous reports from Asia [12]. The prevalence of ESBL-positive isolates in our cohort was relatively high (36.6%) compared with other reports [10,15–17]. This difference may reflect both the temporal trend of rising ESBL rates in more recent studies, as our data covered 2019–2023, and the greater severity of illness and comorbidities in our patient population. We found that ESBL positivity was associated with a greater burden on the healthcare system, as reflected by the longer median length of hospital stay in the ESBL-positive group, consistent with previous reports. In contrast, the in-hospital mortality, the rate of clinical resolution within 72 h, and the occurrence of organ dysfunction did not significantly differ between the ESBL-positive and ESBL-negative groups. The absence of a significant difference may be attributed to two key factors that distinguish our findings from other studies: first, all cases in our study underwent biliary drainage, and second, there was a higher rate of carbapenem antimicrobial use for empirical therapy [11,12,18]. On the other hand, the frequent use of carbapenems could have contributed to the relatively high prevalence of resistance observed in our cohort, underscoring the need for more targeted approaches to antimicrobial stewardship.

Microbiological findings revealed that *E. coli* and *K. pneumoniae* were the predominant pathogens in acute cholangitis cases, with notable differences between the ESBL-positive and ESBL-negative groups. Resistance patterns showed universal resistance to ceftriaxone and cefotaxime among ESBL-positive isolates, whereas carbapenem resistance was higher in ESBL-positive isolates, particularly in *K. pneumoniae* (up to 32.7%). Despite these resistance patterns, ceftriaxone was the most used empirical therapy (45.2%), followed by piperacillin-tazobactam (23.1%) and meropenem (17.5%). This mismatch between empirical antibiotic choices and resistance profiles may have contributed to the rising burden of resistance observed in our cohort, underscoring the importance of tailoring empirical therapy to local susceptibility patterns.

Our study developed a scoring system incorporating both clinical and laboratory predictors of ESBL-associated acute cholangitis, highlighting the role of routine biochemical markers alongside clinical history. While prior antimicrobial exposure and chills are consistent with risk factors highlighted in earlier reports, our analysis also identified hematocrit <27% and ALP > 300 U/L as novel predictors that extend beyond the variables emphasized in previous studies and existing guidelines [6,18,19]. Jang et al. reported age, comorbidities, prior biliary procedures, and antimicrobial use as major contributors to ESBL infection risk [10], whereas the TG2018 guideline primarily stratifies antimicrobial choices by severity of cholangitis [5]. These findings suggest that incorporating routine laboratory markers may provide complementary discriminatory value for risk assessment in addition to established clinical factors. The model demonstrated moderate performance at a cutoff of 7.5 and greater specificity at 11. Given that the model was derived from a single tertiary center with relatively high ESBL prevalence, its transportability to other epidemiological contexts is uncertain and it should be interpreted as a preliminary internally validated risk stratification tool.

Several limitations should also be acknowledged. Bile cultures were obtained after initiation of antimicrobial therapy, which may have reduced microbiological yield and potentially affected detection of antimicrobial resistance, including ESBL-producing organisms. This may have introduced outcome misclassification and could have influenced model performance, predictor selection, and coefficient estimates. The retrospective design raises concerns about the completeness and accuracy of collected variables, as certain exposures (e.g., community-acquired antibiotic use, timing from admission to culture collection, timing of biliary drainage, or detailed procedural histories and a standardized classification of community- versus hospital-acquired cholangitis) may not have been consistently documented. This may have led to residual confounding or underestimation of relevant predictors. In addition, the available data did not reliably capture the temporal sequence and exact timing of key clinical events, precluding time-dependent analyses, which may have further limited our ability to fully characterize the dynamic relationships between treatment, microbiological findings, and clinical outcomes. Moreover, the associations observed should be regarded as hypothesis-generating rather than causal. Low hematocrit may act as a surrogate for sepsis-related anemia or systemic inflammation, while markedly elevated ALP likely reflects significant biliary obstruction, both conditions plausibly linked to more severe infection and repeated healthcare exposure where resistant organisms emerge. Although internal validation was performed, this score was derived from single-center data in a setting with a high prevalence of ESBL-producing organisms, which may limit its generalizability. While bootstrap validation was conducted, external multicenter validation remains essential before the score can be reliably and reproducibly adopted across different epidemiological settings. Future research should focus on prospective validation, inclusion of additional biomarkers, and evaluation of the scoring system’s impact on empirical antibiotic decision-making and stewardship outcomes.

## Conclusion

This study developed an internally assessed preliminary scoring system for predicting ESBL positivity in acute cholangitis using routine clinical and laboratory variables. The system showed moderate performance at a cutoff of 7.5 and greater diagnostic certainty at 11, suggesting potential utility for early risk stratification. At higher cutoffs, the score is best interpreted as a rule-in tool due to its high specificity, whereas its moderate sensitivity at lower cutoffs limits its role as a rule-out strategy. However, any clinical application should be approached with caution and considered in conjunction with local resistance patterns, patient characteristics, and healthcare resources. External validation in multicenter and diverse clinical settings is essential before this scoring system can be recommended for routine clinical adoption. Until such validation is performed, the model should be considered exploratory and hypothesis-generating rather than practice-changing.

## Supporting information

S1 Table Antimicrobial resistance profiles of *Escherichia coli* and *Klebsiella pneumoniae* isolated from blood and bile samples. (DOCX)
