# Supplementary material for: Development and internal validation of a risk stratification scoring system for identifying gram-negative ESBL-positive acute cholangitis: A retrospective cohort study
Source: PLoS One. 2026 Mar 17;21(3):e0345150. doi: 10.1371/journal.pone.0345150 (PMC12994782; doi:10.1371/journal.pone.0345150)
Supplement: S1 Table — (DOCX) [file pone.0345150.s001.docx]

**Table S1**. **Antimicrobial resistance profiles of *Escherichia coli* and *Klebsiella pneumoniae* isolated from blood and bile samples**

| **Antimicrobial agents** | **% of Resistance** | | | |
| --- | --- | --- | --- | --- |
|  | **Non-ESBL** | | **ESBL** | |
|  | ***Escherichia coli*, n=145** | ***Klebsiella pneumoniae,* n=74** | ***Escherichia coli,* n=81** | ***Klebsiella pneumoniae,* n=52** |
| Ceftriaxone | 65 (44.8) | 17 (23.0) | 81 (100.0) | 51 (98.0) |
| Ciprofloxacin | 65 (44.8) | 14 (18.9) | 64 (79.0) | 41 (78.9) |
| Gentamicin | 22 (15.2) | 4 (5.4) | 22 (27.1) | 13 (25.0) |
| Imipenem | 2 (1.4) | 1 (1.4) | 6 (7.4) | 10 (19.2) |
| Meropenem | 1 (0.7) | 0 (0.0) | 7 (8.6) | 13 (25.0) |
| Piperacillin/tazobactam | 1 (0.7) | 0 (0.0) | 11 (13.6) | 22 (42.3) |
| Ertapenem | 1 (0.7) | 0 (0.0) | 9 (11.11) | 17 (32.7) |
| Cefotaxime | 66 (45.5) | 23 (27.0) | 81 (100.0) | 52 (100) |
| Ceftazidime | 65 (44.8) | 20 (31.1) | 81 (100.0) | 52 (100) |
